# High-Definition 3D Exoscope in Pediatric Otorhinolaryngology: A Systematic Literature Review

**DOI:** 10.3390/jcm12206528

**Published:** 2023-10-14

**Authors:** Michele Gaffuri, Antonella Miriam di Lullo, Eleonora M. C. Trecca, Gennaro Russo, Giulia Molinari, Francesca Yoshie Russo, Andrea Albera, Giuditta Mannelli, Massimo Ralli, Mario Turri-Zanoni

**Affiliations:** 1 Research Group of Pediatric Otorhinolaryngology of the Task Force of the Young Otolaryngologists of the Italian Society of Otolaryngology-Head and Neck Surgery, Italy; 2 Department of Otolaryngology—Head and Neck Surgery, Fondazione IRCCS Ca’ Granda Ospedale Maggiore Policlinico, 20122 Milan, Italy; 3 Department of Clinical Sciences and Community Health, University of Milan, 20122 Milan, Italy; 4 Division of Otolaryngology-Head and Neck Surgery, Department of Neuroscience, Reproductive and Odontostomatologic Sciences, University of Naples Federico II, Pansini Street n.5, 80131 Naples, Italy; 5 CEINGE—Advanced Biotechnology, Salvatore G. Street n.486, 80131 Naples, Italy; 6 Department of Maxillofacial Surgery and Otolaryngology, IRCCS Research Hospital Casa Sollievo della Sofferenza, San Giovanni Rotondo, 71013 Foggia, Italy; 7 Department of Otolaryngology, University Hospital of Foggia, 71100 Foggia, Italy; 8 Task Force of the Young Otolaryngologists of the Italian Society of Otolaryngology-Head and Neck Surgery, Italy; 9 Otolaryngology Unit, AORN dei Colli, V. Monaldi Hospital, 80131 Napoli, Italy; 10 Department of Otolaryngology—Head and Neck Surgery, IRCCS Azienda Ospedaliero—Universitaria di Bologna, 40138 Bologna, Italy; 11 Department of Medical and Surgical Sciences (DIMEC), Alma Mater Studiorum—Università di Bologna, 40138 Bologna, Italy; 12 Department of Sense Organs, ENT Department, Sapienza University of Rome, 00185 Rome, Italy; 13 Department of Surgical Sciences, University of Turin, 10124 Turin, Italy; 14 Department of Experimental and Clinical Medicine, University of Florence, 50121 Firenze, Italy; 15 Division of Otorhinolaryngology, Department of Biotechnology and Life Sciences, University of Insubria, 21100 Varese, Italy

**Keywords:** exoscope, exoscopic surgery, surgical procedure, 3-D imaging, pediatrics, otorhinolaryngology, systematic review

## Abstract

This PRISMA-compliant systematic review aimed to investigate the use of and the most common procedures performed with the novel 3D 4K exoscope in surgical pediatric head and neck settings. Methods: Search criteria were applied to PubMed, EMBASE and the Cochrane Review databases and included all studies published up to January 2023 reporting 3D 4K exoscope-assisted surgeries in pediatric patients. After the removal of duplicates, selection of abstracts and full-text articles, and quality assessment, we reviewed eligible articles for number of patients treated, age, surgical procedures, and outcomes. Results: Among 54 potentially relevant records, 5 studies were considered eligible and included in this systematic review, with reported treatment data for 182 patients. The surgical procedures belong to the otologic field (121 cases), head and neck surgery (25 cases) and transoral surgery (36 cases). Exoscopy allowed high quality visualization of anatomical structures during cochlear implantation and during reconstruction in head and neck surgery; moreover, it improved the surgical view of surgeons, spectators and ENT students. Conclusions: The use of 3D 4K exoscopy has shown promising potential as a valuable tool in pediatric ORL-head and neck surgery; nevertheless, further validation of these encouraging outcomes is necessary through larger-scale studies specifically focused on pediatric patients.

## 1. Introduction

In recent years, a groundbreaking advancement in the realm of surgical visualization has emerged in the form of the three-dimensional (3D) 4K exoscope (VITOM^®^; Karl Storz, Tuttlingen, Germany). This innovative tool has swiftly positioned itself as a viable alternative to conventional microscopes and endoscopes, particularly within the specialized field of otorhinolaryngology (ORL) and head and neck surgery [1].

Functioning as a video-telescope, the exoscope harnesses the capabilities of a digital camera system to deliver high-definition images. To fully experience the visual output, 3D 4K high-definition (HD) widescreens and specialized 3D glasses are employed during the surgical procedures. A notable feature of this system lies in its synergy with a mechanical arm known as the ARTip Cruise^TM^ (Karl Storz, Tuttlingen, Germany), which empowers surgeons to actively manipulate the camera, seamlessly adjusting it to any desired angle throughout the course of the surgical intervention.

The integration of this pioneering technology yields a range of technical enhancements when compared to traditional operative microscopes and endoscopes. Among these advantages are the remarkable 3D visualization of anatomical structures, a variable magnification range spanning from 8 to 30 times, an augmented depth of the surgical field spanning from 7 to 44 mm, and a notably extended focal length of 20 to 50 cm. This extended focal length not only provides a wider operative workspace but also augments the depth of focus, facilitating challenging surgical maneuvers [1,2]. Additionally, the widescreen positioning, aligned with the surgeon’s eye level, fosters a more ergonomic working posture compared to the utilization of microscopes and endoscopes. This ergonomic refinement effectively mitigates the physical and mental strain often encountered during surgical procedures, an especially valuable asset during longer and more intricate surgeries [2].

An ancillary benefit arises from the possibility of sharing knowledge and facilitating training. The chance to generate high-definition surgical videos contributes to an enhanced educational environment, enabling effective live and delayed dissemination of surgical techniques to trainees and students [3].

The utilization of the 3D 4K exoscope has found notable application within various facets of adult otorhinolaryngology (ORL) and head and neck surgery. Notably, in the domain of ear surgery, its implementation has encompassed stapedotomy [4], mastoid surgery [5], tympanoplasty [6], and, significantly, the facilitation of cochlear implantation [7]. Case series have surfaced that have detailed exoscope-assisted procedures, such as temporal bone resection [8] and diverse skull base surgeries, spanning both anterior approaches [9] and lateral interventions [10]. Furthermore, reports have emerged documenting its role in transoral surgeries, particularly in the context of procedures such as cleft palate surgery [2], tonsillectomy [3], pharyngoplasty [11], and surgery for obstructive sleep apnea (OSA) [12].

Widening the scope, case series have provided insight into exoscope-assisted thyroidectomy [13], parotidectomy [14], and neck dissection [15], alongside early accounts of microlaryngeal surgery [16]. The outcomes demonstrated in these endeavors have shown favorably promising results when contrasted with conventional visualization tools like microscopes, endoscopes, and surgical loops [15,16]. Such encouraging outcomes have stimulated the call for further comparative clinical investigations, aimed at continuing the exploration of the interplay between established methodologies and innovative approaches. This ongoing comparison seeks to elucidate the genuine value of the exoscope while more precisely defining its attendant advantages and disadvantages.

Despite these promising initial reports and case series, the application of the 3D 4K exoscope within pediatric contexts remains relatively underexplored. In recent years, the literature has witnessed the emergence of numerous case reports focused on the realm of brain surgery [17,18]. However, within the sphere of ORL and head and neck surgery in pediatric cases, only a limited number of published pediatric series have been made available [19,20]. Additionally, case reports have surfaced detailing the application of the exoscope in neck surgery for newborns [21] and children [22], as well as its involvement in cleft palate surgery [23].

This comprehensive review seeks to delve into the realm of exoscope deployment within the context of surgical pediatric head and neck procedures. By examining the array of procedures commonly performed using the exoscope, the study aims to illuminate its influence on surgical outcomes and workflow. Furthermore, the review endeavors to catalog both the advantages and disadvantages of the exoscope in comparison to traditional visualization systems, encompassing operative complications and the overall impact on the surgical landscape.

## 2. Materials and Methods

After registering with the PROSPERO database (ID CRD42023458782), a systematic literature review to evaluate the interest in 3D-exoscopy for pediatric ear, nose and throat (ENT) surgery was performed according to the Preferred Reporting Items for Systematic Reviews and Meta-Analyses (PRISMA) 2020 guidelines. A database search of PubMed, EMBASE and the Cochrane Review of studies published from January 2016 to January 2023 was performed to identify suitable articles. Considering the recent substantial technological advances in 3D-exoscopy, studies published before 2016 were excluded. Records were also identified using additional methods including websites and citation searching.

The PICOS (Participants, Interventions, Comparisons, Outcomes, and Study design) criteria utilized were Participants (P), pediatric patients aged 0 to 17 years, affected by ENT diseases; Intervention (I), surgical treatment using high-definition 3D-exoscopy; Comparator (C), observation; Outcomes (O), surgeon’s comfort, efficacy of the procedure, survival and recurrence rates; Study design (S), retrospective and prospective cohort studies. Relevant keywords, phrases, and medical subject headings (MeSH) database were used according to each database’s requirements. The following is an example of a search strategy used for PubMed/MEDLINE: ((“Exoscope”[Title/Abstract] OR “3D-Exoscope”[Title/Abstract] OR “VITOM-3D”[Title/Abstract] OR “Exoscope 4K Assisted Surgery”[Title/Abstract] OR “Vitom Assisted Surgery”[Title/Abstract] OR “4K 3D-exoscope”[Title/Abstract])) AND ((“Pediatric”[Mesh]) OR ((“Children”[Title/Abstract]) OR (“Newborn”[Title/Abstract])) AND ((“Otorhinolaryngology”[Mesh]) OR ((“ENT”[Title/Abstract]) OR ((“Head and Neck”[Title/Abstract])) AND (2016:2023[pdat]). The “cited by” function on Google Scholar was used to identify additional articles. The last search was performed on 31 January 2023. Two independent authors (A.M.d.L. and M.T.Z.) conducted the electronic search. The following inclusion criteria were considered: full English language text; original articles; studies including clinically confirmed head and neck or skull base pediatric disorders treated with 3D exoscope-assisted surgery; studies reporting detailed surgical information on 4K exoscope-assisted surgery (high-definition images of the surgical field, improved vision and depth perception, different treatment modalities, surgeon’s comfort, and patient’s morbidity). Exclusion criteria were as follows: editorials, letters to the editor, or reviews; studies that included animal samples; non-clinical articles on surgical training and/or cadaver anatomical specimens; studies describing surgical procedures that did not require any surgical field magnification techniques; articles with missing data. After removal of duplicates, all titles and abstracts were evaluated using the inclusion and exclusion criteria. Full texts of the remaining articles were scrutinized in their entirety to determine final eligibility. At the abstract review stage, we included all studies deemed eligible by at least one author. At the full-text review stage, disagreements were resolved by consensus. The included articles were examined for data extraction, including number of patients treated, age, sex, surgical procedures included, outcomes, and follow-up status.

The quality of the included studies was assessed by two authors (E.M.C.T., M.R.) using ‘‘The Strengthening the Reporting of Observational Studies in Epidemiology’’ (STROBE) statement with a score interval from 0 to 22, with a higher score indicating a better study quality. To mitigate the risk of bias, papers of all quality were included in this systematic review.

## 3. Results

**Figure 1:**
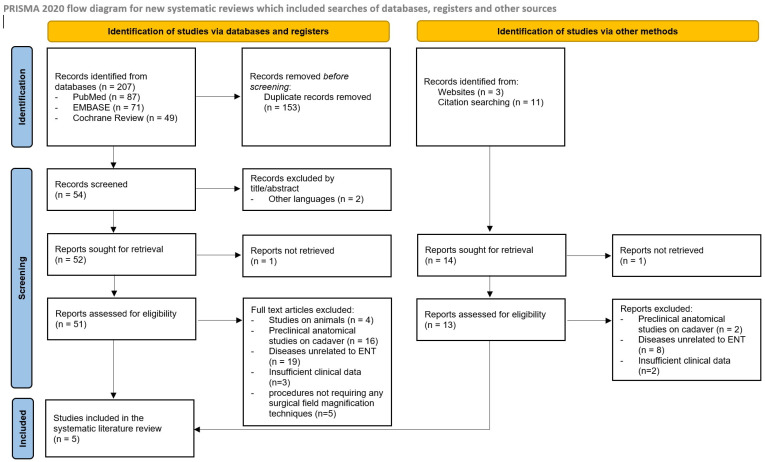
PRISMA flow-chart summarizing the methods used in the present review.

The PRISMA flow diagram is shown in Figure 1. After duplicate removal, 54 potentially relevant records were identified through database searching and other sources. To note, two studies were excluded after title and abstract review and one was not retrieved, while the full text of the remaining fifty-one articles were examined for further review. Finally, five studies were screened for eligibility and included in this systematic review [17,18,19,20,21]. A total of 182 patients were described in the selected studies, as summarized in Table 1. However, all studies were focused on analyzing advantages and disadvantages of the new surgical technique and therefore some clinical data were not available (sex, follow-up). The mean age was between 68 months and 8 years. The surgical procedures belong to the otologic field, head and neck surgery, and transoral surgery.

Regarding otology, a total of 121 cases were performed by exoscopy technique, according to the literature data in English (19 type I tympanoplasty, 7 type II/III tympanoplasty without chronic otitis media (COM), 18 type II/III tympanoplasty with COM, 72 cochlear implantations (CI), 3 middle ear implantations, 2 other ear pathologies). Regarding head and neck surgery, a total of 25 patients were approached by exoscopy (9 laryngotracheal surgery, 4 thyroidectomies, 4 parotidectomy, 2 congenital nasal pyriform aperture atresia, 6 other head and neck pathologies). Regarding transoral surgery, a total of 36 cases were performed by exoscopy (20 velo-palatoplasty, 10 cheiloplasty, 3 plunging ranula, 2 gingivoplasty, 1 tongue resection). These recent data do not allow an adequate follow-up. However, they permit the conclusion that in the otologic field, the exoscopy is less efficient when the surgical cavity is narrow (external auditory canal, EAC, oval window, OW) or when there is inflammatory tissue (COM). On the contrary, the exoscopy enables high quality visualization (stereoscopy, lighting, and focusing) during CI concerning both posterior tympanostomy and electrode insertion in over 80% of cases. Conversely, regarding head and neck surgery and transoral surgery, the exoscopy allows a clear improvement in identifying anatomical structures and obtaining better reconstructions (nerves and vessels).

In one of the studies eligible for review [20], immediately after each surgery, the surgeon completed a survey to evaluate differences between the exoscope and the traditional microscope or surgical loupes, estimating the contribution of each of these solutions on a scale of 0 to 100: for otologic surgeries, the mean scores (/100) for the contribution of the exoscope compared to the microscope were 68.4 (±23.2); for transoral and cleft palate surgery, the mean score (/100) for the contribution of the use of the exoscope compared to the magnifying loupes was 92.9 (±8.6); meanwhile, for open head and neck surgeries, the mean score (/100) was 89.5 (±7.2).

In the same study [20], operating room occupancy times for surgeries performed under the microscope (n = 18) and under the 3D-exoscope (n = 41) were compared, considering the standardization of cochlear implant surgeries: no significant difference was observed between the two groups: 210.8 (±52.2) minutes for the microscopy group and 211.7 (±50.3) minutes for the exoscope group (*p* value = 0.95).

In addition to these technical advantages, the exoscopy improves the surgical view of surgeons, spectators, and ENT students’ learning by a wider operating field with an overview on fine and deep dissection through 3D-glasses obtaining significant ergonomic and teaching benefits. No technical difficulties or complications were reported in these cases.

The assessment process revealed that the included studies were mostly of very high quality (STROBE score: 19.6 ± 1.8). Lower scores were mainly due to the number of participants, the descriptive data provided, the analysis of variables and limitations, the generalizability, and the presence of bias.

## 4. Discussion

This systematic review, which focuses on the use of the 3D 4K exoscope in pediatric otorhinolaryngology, highlights the increasing interest in this innovative technology and its application in ORL-head and neck surgery for children. Recent advancements in 3D exoscopy have resulted in larger case series being published in recent years [19,20], preceded by case reports involving challenging clinical conditions [21,22,23] that were successfully treated with exoscope assistance.

The review reports a substantial number of patients treated with the 3D 4K exoscope in a relatively short period (181 cases reported), supporting its efficacy in pediatric treatment. Most of the included studies demonstrated high methodological quality. However, the lack of data from randomized controlled trials prevents definitive conclusions regarding the use of this novel technology for pediatric ORL-head and neck surgery.

The primary advantage of the 3D 4K exoscope is its ability to provide a magnified, wider, and deeper operative view, enabling visualization and navigation of the surgical field in three dimensions. This feature is particularly valuable in cases with limited access and small dimensions, where there is a high risk of nervous or vascular injuries. For instance, Chebib et al. [20] described the successful use of the 3D 4K exoscope in 35 patients undergoing uvulopalatoplasty, while Meier et al. [23] reported positive outcomes in a case report of cleft palate repair. Similar positive outcomes were observed in head and neck surgery, specifically in laryngotracheal procedures, where the exoscope facilitated anatomical structure identification and improved reconstruction of nerves and vessels [21,22]. These findings are consistent with prior reports on the application of exoscope-assisted surgery in adult patients: case studies involving various oncological procedures, including demolition and reconstruction, have been documented. For instance, De Virgilio et al. [24] shared their experience with free flap harvesting and microvascular anastomosis in adult patients undergoing head and neck reconstruction surgery: this report described successful arterial and venous anastomoses without complications in multiple cases. In this study, the conventional operative microscope commonly used in this field was substituted with the exoscope. Furthermore, a recent study [25] highlighted the use of a 3D exoscope for transoral excision of a soft palate tumor, as well as free flap harvesting and subsequent insetting for intraoral defect reconstruction. Microvascular anastomosis was also performed, underscoring the utility of this innovative visualization technique in transoral surgery. This approach enabled precise suture placement, which is crucial for preventing suture dehiscence and potential fistula formation. Notably, the main advantage of an easy handling of surgical instruments and precise execution of challenging surgical maneuvers within the confined space of the oral cavity has been proved particularly effective in pediatric patients.

Despite these promising preliminary results, otological procedures for conditions like COM or EAC stenosis have not shown the same level of efficacy using the exoscope. Surgeons reported that the exoscope was less efficient than the microscope in approximately 30–35% of cases for the identification of anatomical structures, lighting and depth of the surgical field, and camera handling. Additionally, in 47% of cases, the exoscope provided inferior magnification compared to the microscope [20]. This discrepancy may be attributed to the exoscope’s performance in inflammatory environments or narrow operating fields, in the presence of poor lighting in small surgical corridors and when high magnifications are required, with potential deterioration of the surgical images with consequent pixelization [20,26,27,28]. In contrast, the 3D 4K exoscope has demonstrated satisfactory performance during CI [19,20], offering high-quality visualization during posterior tympanotomy and electrode insertion, with only the occasional need for switching to the microscope. Moreover, no additional complications have been reported in the literature when compared to traditional otologic surgery, and this finding is the same as for adult patients [1].

Currently, there exists a lack of published studies assessing the viability of utilizing the 3D 4K exoscope in microlaryngeal surgery as a substitute for the conventional operating microscope or endoscope within the context of pediatric patients. Limited case series involving adults have been documented [16,29]. In a recent series involving 41 consecutive cases, the exoscope was employed in therapeutic (75.6%) and diagnostic (24.4%) procedures: notably, all surgeries were completed successfully without the need for the operative microscope, and no instances of complications or delays were identified [16]. Following each procedure, the surgical team, including the surgeon and scrub nurse, were prompted to complete a tailored questionnaire employing a 3-point Likert scale (1—not acceptable, 2—acceptable, 3—good). This questionnaire comprised 12 specific items. A noteworthy portion of these individual items were rated as “good” by both the surgeons (81.1%) and scrub nurses (87.5%). Particularly highly rated aspects included the natural ergonomic posture maintained during the procedure, as well as the ease of utilizing the joystick and adjusting focus, as assessed by the surgeons.

These advantageous features of the exoscope are likely applicable within the realm of pediatric microlaryngeal surgery, alongside various other benefits. Notably, the exoscope presents an enhanced visual experience, offering higher magnification and a 3D 4K depiction of laryngeal structures, surpassing the conventional microscopic or endoscopic views. This heightened visualization potential holds the promise of facilitating more precise and symmetrical laryngeal modifications, a critical aspect, for instance, during procedures like supraglottoplasty for laryngomalacia. Such precision aids in averting discrepancies between the two sides of the larynx, which is particularly crucial for preventing irregular scarring, especially in the delicate context of newborns and pediatric patients. Furthermore, the exoscope can be seamlessly integrated with a CO_2_ laser, affording a heightened degree of precision during cutting procedures and minimizing instances of bleeding [29].

The previously cited enhanced ergonomics of the exoscope offers significant advantages to surgeons by projecting images onto a 3D 4K widescreen positioned at eye level. This configuration diminishes both physical strain and mental stress, thereby alleviating potential frustration. In a study conducted by Tewfik et al. [2], a comparison was made between performing cleft palate surgery on a cadaveric specimen using an endoscope and an exoscope. The assessment was carried out employing a NASA Task Load System (TLS) questionnaire [30], a well-established multidimensional scale that yields an overall workload score. This score is calculated based on a weighted average of six subscales, encompassing mental demand, physical demand, temporal demand, performance, effort, and frustration. Both the endoscope and exoscope facilitate an upright posture and reduced physical effort, attributes that were corroborated by the outcomes of the NASA TLS questionnaire; in particular, the results of this study indicated a superior score for the exoscope in contrast to the endoscope: participants’ responses indicated that the use of the exoscope entailed lower levels of intellectual, physical, and temporal demands compared to the endoscope. Moreover, the exoscope was associated with reduced efforts and perceived frustration during video-assisted procedures. Furthermore, the overall surgical performance was regarded as less demanding and of higher quality when utilizing the exoscope. The overall comfort provided by this innovative visualization technique could be particularly pivotal in the context of pediatric surgery, due to the delicate nature of pediatric patients, such as newborns and infants, coupled with the challenge of navigating smaller and consequently more intricate anatomical spaces. In such scenarios, the objective remains to minimize both physical and mental strain, underscoring the importance of optimal comfort and ease of use.

The transition between conventional visualization tools and exoscope has not been reported as difficult: Chebib et al. [20] noted that the seamless transition between exoscope and direct vision is facilitated by the small size of the camera, and the use of glasses does not impede direct vision of the surgical field, unlike virtual reality glasses. These features, which have been well-documented in the literature for adult patients, hold true for pediatric patients as well. However, excessive visual fatigue due to the use of polarizing glasses for the 3D view for longer procedures has been linked to headaches and dizziness affecting surgeons [29,31,32]. Further studies on these aspects are needed to confirm this funding also in pediatric patients, as no manuscripts were found in the literature dealing with this situation.

Thanks to this novel technology, the surgical images can be shared with the entire operating room, facilitating knowledge transmission to trainees and students [32]. The exoscope provides 4K images on large widescreens, ensuring ideal vision not only for operators but also for all participants. This feature allows the exercises and/or surgical steps to be shown clearly and effectively to all participants and the evaluation of performances by a senior surgeon. Several reports have been published so far about the use of the exoscope for training in microvascular surgery [33], microneurosurgery [34], ear surgery and temporal bone dissection [32], and tonsillectomy [3], making this technology a useful tool for live and delayed surgical training. This advantage could be exploited especially for training and shadowing pediatric ORL-head and neck surgeons.

As a final consideration, it is possible to state that the 3D 4K exoscope is filling the void left by robotic surgery in pediatric patients. Up to now, the field of robotic surgery in children has been applied to abdominal, thoracic, urological, and gynecological procedures. Due to its success in other fields, researchers began to investigate its use in otolaryngology procedures. In 2007, the first feasibility study for the application of transoral robotic surgery (TORS) in pediatric airway procedures was successfully conducted [35]. Since then, TORS has been successfully applied for use in pediatric lingual tonsillectomy, laryngeal cleft repair, cricoid split of interarytenoid and transglottic scar tissue, partial arytenoidectomy with cordectomy, thyroglossal duct cyst marsupialization, airway lymphatic malformations resection, and tongue reduction [36], but these reports represent off-label use and contain small sample sizes. Recently, a case series on lingual thyroglossal duct cyst robotic removal has been published [37], thanks to the anatomical concept that the relatively avascular channel of the midline posterior tongue, vallecula, and posterior hyoid space provides a safe plane of dissection for deep lesions of the tongue and access to structures in the anterior neck. Moreover, in the literature there are a few additional reports about robotic thyroidectomy through transaxillary [38] or transhairline approaches [39]. The main limitations on the use of robots in transoral surgery are the small size of the pediatric oral cavity and the characteristically bulky arms of robotic surgical systems, which when combined, result in crowding and bumping of the arms [35,36]. As robotic surgeons gain experience, application of this technology will continue to grow, but until this novel technology can be adapted to smaller dimensions such as in children and is officially approved for pediatric patients, 3D 4K exoscopy could help surgeons to overcome limits of the traditional naked, microscopic and/or endoscopic surgical view.

In the context of this systematic review, we made concerted efforts to minimize bias during the article selection and data extraction processes. However, there are some notable factors that could introduce publication bias. Specifically, the inclusion of case reports, coupled with the absence of relevant randomized controlled trials and large-scale observational studies for comparing the exoscope with traditional microscope/endoscope approaches, may tilt the results towards significance. Nevertheless, this inclusion bias was deemed necessary due to the limited existing literature on this innovative technology.

Another limitation of this study stems from the clinical heterogeneity of the included studies, which makes it impossible to objectively assess therapeutic success. It is essential to acknowledge that, while various studies have reported promising results and no significant disparities between the exoscope and conventional visualization methods, caution must be exercised when interpreting the data. This caution is warranted because the number of enrolled patients across these studies varies significantly.

To address these limitations, future research should prioritize more consistent reporting and focus on the need for larger and more robust patient cohorts. This would enable a more comprehensive evaluation of the exoscope’s performance in comparison to traditional visualization techniques.

## 5. Conclusions

The use of 3D 4K exoscopy has shown promising potential as a valuable tool in pediatric ORL-head and neck surgery. Its application has been observed in otologic, transoral, and cervical surgeries, where it has proven to be effective and safe without any additional complications compared to traditional surgical methods. Moreover, good results obtained in microvascular and reconstructive surgery in adult patients could be reproduced in children, especially in challenging procedures for pathologies of the oral cavity, one of the narrowest and most difficult-to-access spaces of the head and neck district. Other adjunctive pediatric surgical fields need to be explored in relation to the use of the exoscope, in particular laryngeal and airway surgery, where technical advantages, such as the 3D and HD visualization, could help surgeons in performing precise, symmetrical, and safe procedures, such as supraglottoplasty for laryngomalacia. Also, thanks to the availability of a combined simultaneous use of a CO_2_ laser, the exoscope affords a high degree of precision during cutting procedures and in minimizing bleeding.

The transition between conventional visualization tools (such as surgical loupes, operative microscope, and endoscope) and the exoscope has been reported as being relatively easy, as direct vision is facilitated by the small size of the camera, and the use of glasses does not impede direct vision of the surgical field, even if excessive visual fatigue due to the use of polarizing glasses for the 3D view in long lasting procedures has been linked to sporadic headaches and dizziness. The exoscope favors enhanced ergonomics and the upright posture of the surgeons, thanks to the significant advantage of projecting images onto a 3D 4K widescreen positioned at eye level. This configuration diminishes both physical strain and mental stress, thereby alleviating perceived frustration and enhancing the overall surgical performance; these results could be fundamental in the context of neonatal and pediatric surgery, helping surgeons in the challenge of navigating smaller and consequently more intricate anatomical spaces. Additionally, this novel technology offers educational benefits thanks to the opportunity of live and delayed teaching for students and trainees, allowing an effective explanation of exercises and surgical steps, a precise evaluation of performances by a senior surgeon, and a consequent efficient shadowing of pediatric ORL-head and neck surgeons, in addition to being an easy way to perform surgical training on human, animal, and artificial models.

While awaiting the development of officially approved and pediatric-specific robotic surgery options, which have so far been successfully applied off-label in procedures involving the mouth, airway, and neck, the use of 3D 4K exoscopy presents an opportunity for surgeons to surpass the limitations of traditional surgical views. Nevertheless, further validation of these encouraging outcomes is necessary through larger-scale studies specifically focused on newborns and children.

## Figures and Tables

**Table 1:** Review of the literature and analysis of described cases.

Author, Year	No. of Cases	Mean Age	Sex	Surgical Procedures	Outcomes	Follow-Up	STROBE Score *
***Chebib, 2022* [20]**	151	68 months	Female 72 (48%) Male 79 (52%)	**Otology 93 (62%)** Type I tympanoplasty 19 (21%) Type II/III tympanoplasty (no COM) 7 (8%) Type II/III tympanoplasty (COM) 18 (19%) Cochlear implantation 44 (47%) Middle ear implantation 3 (3%) Others (External auditory canal cholesteatoma and temporal bone tumor) 2 (2%)	**Otology:** EX is less efficient when the surgical field is narrow (EAC, OW) or if the tissues are inflammatory (COM)	NA	22
				**Head and neck surgery 23 (15%)** Open laryngotracheal surgery 7 (31%) (excluding standard tracheostomy) Thyroidectomy 4 (17%) Parotidectomy 4 (17%) Congenital nasal pyriform aperture atresia 2 (9%) Others (rhinoseptoplasty—2, infratemporal tumor resection—2, nasal dermoid sinus cyst—1, cervical teratoma resection—1) 6 (26%)	**H&N Surgery and transoral surgery:** the position is not conditioned by the depth of the surgical field, the results satisfactory, a clear improvement to identify anatomical structures allowing for better reconstruction (nerves and vessels).		
				**Transoral surgery 35 (23%)** Velo(-palato)plasty 19 (54%) Cheiloplasty 10 (28%) Plunging ranula 3 (9%) Gingivoplasty 2 (6%) Tongue resection 1 (3%)			
***Chebib, 2021* [21]**	1	10 days	NA	Closure of H-type TEF by two layers (one tracheal and one esophageal) using a right cervical approach	Improvement in the surgical view of surgeons, spectators, and ENT students’ learning by a wider operating field with a clear overview on fine and deep dissection through 3D-glasses.	NA	18
***Chebib, 2021* [22]**	1	6 years	NA	PCTR for acquired SGS (grade III according to Myer-Cotton classification)	Improvement in the surgical view of surgeons, spectators, and ENT students’ learning by a wider operating field with a clear overview on fine and deep dissection through 3D-glasses.	NA	18
***Rusetsky, 2022* [19]**	28	4.5 years	Female 12 Male 16	Cochlear implantation	No technical difficulties or complications. EX enabled high-quality visualization (stereoscopy, lighting, and focusing) during both posterior tympanotomy and electrode insertion. The chief surgeon evaluated the quality of surgical images very highly in over 80% of cases.	NA	21
***Meier, 2022* [23]**	1	8 years	NA	Cleft palate repair	Significant ergonomic and teaching benefits.	NA	19

NA: not available; EX: exoscope; EAC: external auditory canal; OW: oval window; COM: chronic otitis media; H&N surgery: head and neck surgery; TEF: trachea–esophageal fistula; PCTR: partial cricotracheal resection; SGS: subglottic stenosis. * Scores interval from 0 to 22, with higher scores showing better study quality.

## Data Availability

All data pertaining to this systematic review are available from the corresponding author upon reasonable request.
